# CD68 as a multi-omic prognostic biomarker in digestive system cancers: correlations with tumor-infiltrating immune cells and immune checkpoints

**DOI:** 10.3389/fimmu.2025.1599677

**Published:** 2025-08-21

**Authors:** Hui Li, Hui Zhang, Rujiang Dai, Dian Zheng, Jianghai Zhao, Hong Jing, Xuhui Ma, Lin Zhang, Weihong Sun, Zhimin Suo

**Affiliations:** ^1^ Department of Digestion, Huaihe Hospital of Henan University, Kaifeng, China; ^2^ Department of Pathology, Huaihe Hospital of Henan University, Kaifeng, China; ^3^ Department of Medical Equipment, Huaihe Hospital of Henan University, Kaifeng, China

**Keywords:** CD68, digestive system cancers, macrophage, prognosis, biomarker, immune checkpoint

## Abstract

**Background and objective:**

CD68 plays a crucial role in promoting phagocytosis. However, its expression level, prognostic value and the correlations with tumor-infiltrating immune cells (TIICs) or common tumor immune checkpoints (TICs) in human digestive system cancers (DSC) remain poorly understood. This study aims to investigate the expression levels, prognostic significance, and clinical implications of CD68, as well as its correlations with six TIICs and four common TICs in DSC.

**Materials and methods:**

We analyzed CD68 mRNA and protein expression using online databases and immunohistochemistry (IHC) on tissue microarray (TMA) sections, comparing DSC tumor tissues with adjacent normal tissues. Overall survival (OS) was calculated to evaluate the prognostic value of CD68 in DSC. Additionally, correlations between CD68 expression and six TIICs (B cells, CD4+ T cells, CD8+ T cells, macrophages, NK cells, and cancer-associated fibroblasts) or four common TICs (PDCD1, CTLA4, IDO1, and CD40) were assessed using the Tumor Immune Estimation Resource (TIMER).

**Results:**

CD68 mRNA expression was significantly higher in esophageal carcinoma (ESCA) and stomach adenocarcinoma (STAD) tissues compared to adjacent normal tissues, but lower in colon adenocarcinoma (COAD), liver hepatocellular carcinoma (LIHC), and pancreas invasive ductal carcinoma (PAAD). Protein expression of CD68 was significantly higher in COAD than in adjacent normal tissues, but lower in ESCA, LIHC, PAAD, and STAD. CD68 protein expression served as a prognostic marker in COAD and STAD. Furthermore, CD68 expression showed strong positive correlations with the six TIICs and significant positive correlations with the four TICs in DSC.

**Conclusion:**

CD68 may serve as an essential prognostic biomarker in COAD and STAD and could be a promising candidate for diagnostic, prognostic, and therapeutic targeting in human DSC.

## 1 Introduction

Digestive system cancers (DSC), including esophageal carcinoma (ESCA), stomach adenocarcinoma (STAD), colon adenocarcinoma (COAD), liver hepatocellular carcinoma (LIHC), and pancreas invasive ductal carcinoma (PAAD), are among the leading causes of cancer-related morbidity and mortality worldwide (1). Despite the increasing diversity of cancer treatment options, the mortality rate of DSC remains high. In recent years, the tumor microenvironment (TME) has attracted significant attention as a therapeutic target in cancer research and clinical practice (2, 3). The TME plays a critical role in cancer progression, with tumor-infiltrating immune cells (TIICs) acting as key mediators of immune responses and tumor behavior (4). Among these, macrophages, marked by the expression of CD68, are central to phagocytosis, immune regulation, and tumor progression (5). The TME consists of tumor cells, tumor-associated fibroblasts, immune cells, and other components. Tumor-associated macrophages (TAMs) exhibit significant heterogeneity within different TMEs, exerting diverse functions through various subtypes and thereby influencing tumor progression in distinct ways (6). This suggests that TAMs may serve as valuable markers for monitoring cancer prognosis. Therefore, we aimed to identify a prognostic biomarker that could help explain the heterogeneity of the TME in DSC.

CD68, a glycoprotein predominantly expressed in macrophages, has been implicated in various cancers due to its role in modulating immune responses and tumor-associated inflammation (7). However, the expression patterns, prognostic significance, and interactions of CD68 with TIICs and immune checkpoints in DSC remain poorly understood. The total amount of TAMs is often used to evaluate their correlation with cancer progression, and CD68 is a general macrophage marker for quantifying total TAM levels (8). CD68 is a heavily glycosylated transmembrane glycoprotein that is highly expressed in human tissue macrophages and monocytes (9). As a myeloid-specific surface marker, CD68 is particularly highly expressed in macrophages (10). Studies have reported that overexpression of macrophage antigens in tumor tissue may indicate a prometastatic state and could be associated with poor prognosis (10–13). For instance, previous research has shown that TAM infiltration positively correlates with tumor cell proliferation in breast carcinomas (11–14). Additionally, the expression level of CD68 is generally higher in tumor tissues compared to normal tissues (15). and a high density of CD68 predicts poor prognosis in STAD CD68 was significantly higher in COAD than in normal tissues and evaluating macrophage infiltration has clinical value in developing individualized treatment plans for COAD patients (16). Kui Yang et al.’s research indicates that high levels of CD68 in tumor samples correlated with an adverse prognosis in LIHC (17).

Tumor immune checkpoints (TICs) such as PDCD1 (PD-1), CTLA4, IDO1, and CD40 are critical regulators of immune responses and have emerged as promising therapeutic targets in cancer immunotherapy (18). Understanding the relationship between CD68 expression and these TICs could provide valuable insights into the immune landscape of DSC and identify potential biomarkers for diagnosis, prognosis, and therapy.

In this study, we aim to elucidate the expression levels of CD68 in various DSC types, assess its prognostic value, and explore its correlations with six TIICs (B cells, CD4+ T cells, CD8+ T cells, macrophages, NK cells, and cancer-associated fibroblasts) and four common TICs (PDCD1, CTLA4, IDO1, and CD40). By leveraging online databases and immunohistochemical analysis of tissue microarrays (TMAs), we seek to determine whether CD68 can serve as a prognostic biomarker and a potential therapeutic target in DSC.

## 2 Materials and methods

### 2.1 Data mining of CD68 mRNA expression in DSC by TGCA database

The mRNA expression of CD68 in cancerous and normal tissues in DSC was analyzed by (https://tnmplot.com/analysis/) (19, 20) which containing paired cancerous and normal human tissues. We detected the differential expression between paired cancerous and normal tissues for CD68 across The Cancer Genome Atlas tumor (TCGA) by using RNA-Sequencing data. The mRNA expression of CD68 was displayed using box plots, showing with the median, spread and outliers by RNA-Sequencing normalized by transcript per million (TPM) across normal and cancerous tissues.

### 2.2 Patients and tissue specimens

All samples were obtained from patients with DSC who had surgery in Huaihe Hospital of Henan University. This research was authorized by the Ethics Committee of Huaihe Hospital of Henan University, and written informed consent was obtained from each patient. All the specimens analyzed were anonymized. All cases were diagnosed histologically according to the World Health Organization classification. The data were obtained from the medical records, pathology reports, and hospital follow-up records.

### 2.3 TMAs construction

All tissues were fixed in 4% buffered formaldehyde and embedded in paraffin for constructing TMAs. One core (1mm diameter) was removed from each paraffin embedded sample and inserted into a blank paraffin block. One TMA section was comprised of up to 120 cores. Two separate TMAs were made, containing 5 kinds of different digestive system cancers. TMA blocks were cut at a 4μm thickness, which were mounted on microscope slides.

### 2.4 Immunohistochemistry and evaluation

IHC was used to detect CD68 expression in TMAs of human DSC specimens. The detailed IHC protocol was available in our previous article (21). Briefly, paraffin-embedded TMA sections were deparaffinized in xylene, rehydrated in graded ethanol solutions, and the endogenous peroxidase activity was blocked by incubation with 3% H_2_ O_2_ for 30 min at room temperature. Then, the sections were immersed in a citrate-NaOH buffer (10 mM sodium citrate, pH 7.0) for 40 min at 92°C to restore antigenicity. The rehydrated sections were incubated overnight at 4°C with the rabbit anti-human CD68 polyclonal antibody (1:50, ABclonal, No.: A13286, USA). Then the sections were washed with Tris-buffered saline (TBS) and incubated with the MaxVision TM HRP-Polymer anti-Rabbit IHC Kit (Maixin, Fuzhou, China) for 15 min at room temperature. The sections were visualized using the DAB Detection Kit (Maixin, Fuzhou, China), and the reaction was followed by counterstaining with hematoxylin. The negative control experiments were performed by omitting the primary antibody.

IHC slices images were scanned using a ScanScope T2 automated slide-scanner (Aperio Technologies, Vista, CA, USA), and the histochemistry Score (H-score) was evaluated by a pathologist blinded to clinical and molecular data using H-scores (22) ([H-score=∑(pi×i) = (percentage of weak intensity area×100) +(percentage of moderate intensity area×200) +(percentage of strong intensity area×300).]), in which “pi” represented the percentage of positive cells among all cells in the various intensity categories, and “i” represented the staining intensity. H-score is a value between 0-300, the larger the value, the stronger the overall positive intensity.

### 2.5 Survival analysis

Overall survival (OS) was computed from the date of patients with DSC who had surgery to the date of death or the last follow-up and the KM plotter database (https://www.kmplot.com/analysis/). We explored the expression of CD68 on OS in above 5 digestive system cancers. Survival analyses were carried out to achieve Kaplan-Meier survival curve (KM curve). Hazard ratios (HRs) with 95% confidence intervals (95%CIs) and log-rank P-values were calculated.

### 2.6 Estimation of correlations between CD68 expression and six TIICs (B cells, CD4+ T cells, CD8+ T cells, macrophages, NK cells and cancer associated fibroblast) in DSC via TIMER

We investigated the relationships between CD68 expression and the number of six TIICs (B cells, CD4+ T cells, CD8+ T cells, macrophages, NK cells and Cancer associated fibroblast) in COAD, ESCA, LIHC, PAAD and STAD by using TIMER (23–25) database. We selected the tumor “Purity” to adjust our analysis. The partial spearman’s association analysis was used to determine the correlation coefficient.

### 2.7 Exploration of correlations between CD68 expression and four common TICs (PDCD1, CTLA4, IDO1 and CD40) in DSC by using TIMER

We explored the correlations between CD68 expression and four common TICs (PDCD1, CTLA4, IDO1 and CD40) in COAD, ESCA, LIHC, PAAD and STAD by using TIMER. The partial spearman’s association analysis was used to determine the correlation coefficient.

### 2.8 Multiplex Immunofluorescence in COAD/STAD samples

To investigate macrophage heterogeneity and immunosuppressive niches in COAD and STAD, we performed tyramide signal amplification (TSA)-based multiplex immunofluorescence (mIF) for CD68 (pan-macrophage marker), CD163 (M2 marker), iNOS (M1 marker), and PD-L1 on formalin-fixed, paraffin-embedded (FFPE) tissue samples. Following antigen retrieval, sections were sequentially incubated with primary antibodies against CD68 (1:500, Aifang Biological, AF20022), CD163 (1:500, Aifang Biological, AF20010), iNOS (1:400, Aifang Biological, AFRP0001), and PD-L1 (1:300, Aifang Biological, AF20084), followed by fluorophore-conjugated secondary antibodies and TSA amplification with inter-cycle stripping. Nuclei were counterstained with DAPI. Images were acquired using an 8-channel fluorescence whole slide scanner (AF-KL-20-8, 40× objective) and analyzed with K-Viewer software (v1.0.5) for spectral unmixing and quantification.

### 2.9 Statistical analysis

The Mann-Whitney test was performed to analyze CD68 mRNA expression. The data of CD68 IHC was analyzed with GraphPad Prism5 and presented as the means ± SD. Statistical significances were calculated with T-test. The survival conditions was analyzed by KM curves, the log-rank test and the Cox proportional hazards regression model. The correlation of gene expression and TIICs or TICs was evaluated using partial Spearman’s correlation analysis. Differences were considered significant for P < 0.05.

## 3 Results

### 3.1 The mRNA expression of CD68 in human DSC

**Figure 1:**
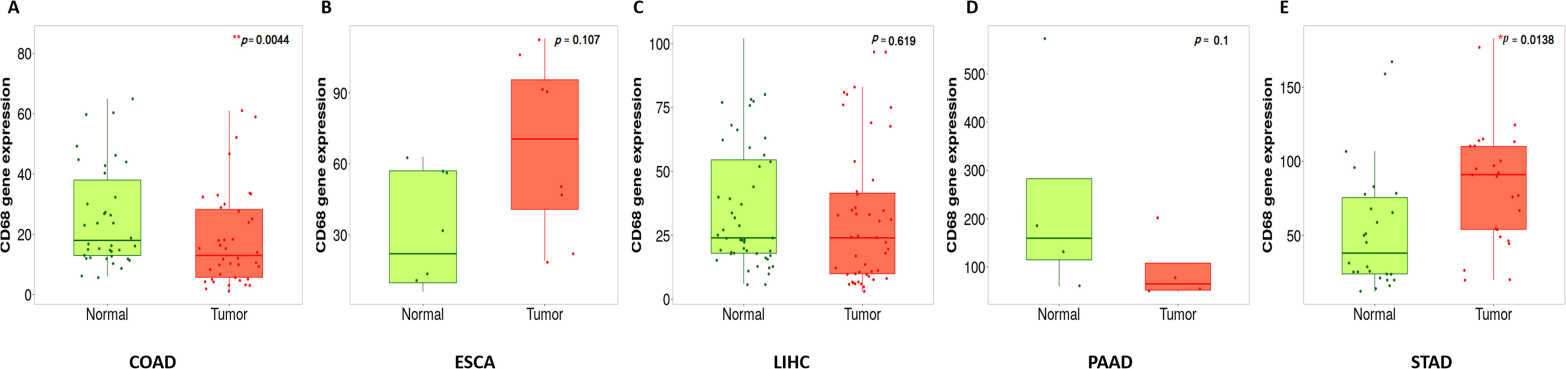
Boxplots of CD68 gene expression in human DSC compared with paired normal tissues by using gene array data. **(A)**, CD68 expression in COAD (n=41paired), **p=0.0044. **(B)**, CD68 expression in ESCA (n=8paired), ns, *p*=0.107. **(C)**, CD68 expression in LIHC (n=80paired), ns, *p*=0.619. **(D)**, CD68 expression in PAAD (n=4paired), ns, *p*=0.1, **(E)**, CD68 expression in STAD (n=27paired),*p=0.0138. The comparison of CD68 expression in pared tumor and adjacent normal tissues was used by running a paired Wilcoxon statistical test.

We investigated mRNA expression of CD68 in DSC using TGCA database in . The results revealed that mRNA expression of CD68 was higher in cancer tissues in ESCA (ns, no significant. *p*=0.107) (**Figure 1B**) and STAD (*, *p*=0.0138) (**Figure 1E**), but lower in COAD (**, *p=*0.0043) (**Figure 1A**), LIHC (ns, *p*=0.619) (**Figure 1C**) and PAAD (ns, *p*=0.1) (**Figure 1D**), each compared to normal tissues.

### 3.2 CD68 protein expression in DSC

**Figure 2:**
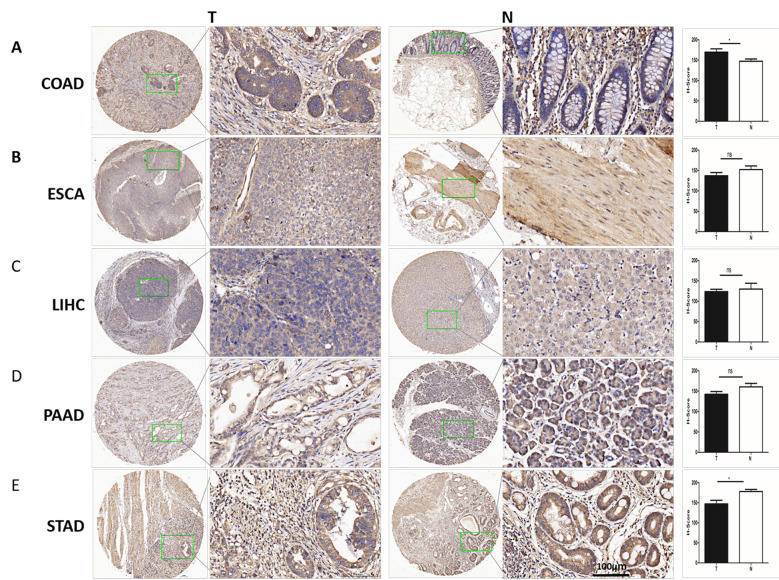
The protein expression of CD68 in human DSC tissues compared with normal tissues. **(A)** The protein expression of CD68 was significantly higher in COAD (n=24paired) than in its adjacent normal tissues(*). **(B)** The protein expression of CD68 was lower in ESCA (n=24paired) than in its adjacent normal tissues (ns). **(C)** The protein expression of CD68 was lower in LIHC (n=24paired) than in its adjacent normal tissues (ns). **(D)** The protein expression of CD68 was lower in PAAD (n=24paired) than in its adjacent normal tissues (ns). **(E)** The protein expression of CD68 was significantly lower in STAD (n=24paired) than in its adjacent normal tissues(*). The quantification of IHC was formalized into mean optical density detected by Image-Pro Plus2.0. Bars show the means ± SD. Difference was statistically significant (*p < 0.05). Original magnification: 200×. Scale bar: 100 μm.

According to the IHC results. We found that the protein expression of CD68 was significant higher in COAD (*) than in its adjacent normal tissues (**Figure 2A**), but was lower in ESCA (ns.), LIHC(ns.), PAAD(ns.) and STAD (*) than in their each adjacent normal tissues (**Figures 2B-E**). Additionally, The CD68 protein expression was observed only in cytoplasm in COAD, ESCA, LIHC and PAAD (**Figures 2A-D**), and in both nuclei and cytoplasm in STAD (**Figure 2E**).

### 3.3 Expression of CD68 can serve as prognostic marker in COAD and STAD

**Figure 3:**
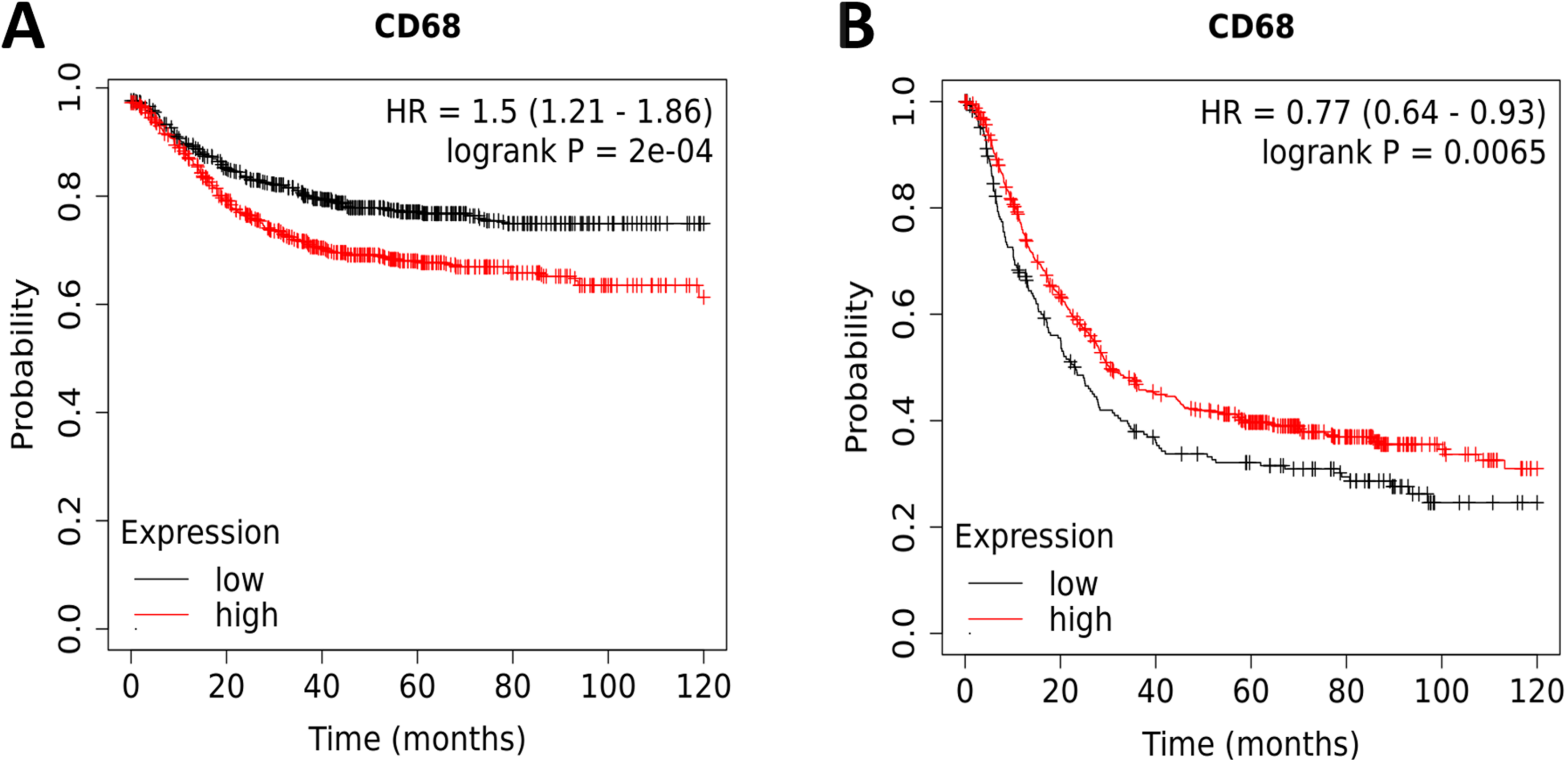
Kaplan-Meier survival curves comparing the high and low protein expression of CD68 in COAD and STAD. **(A)** OS of COAD (n=1336), **(B)** OS of STAD (n=875). Red curve indicates patients with high expression of CD68, while black curve indicates patients with low expression of CD68. HRs with 95%CIs and log-rank P-values were calculated and shown in each curve. P < 0.05 was considered statistically significant.

We proceeded to determine whether the expression of CD68 protein is associated with the prognosis of patients in DSC. The results of OS were as follows: COAD (OS: HR = 1.5, 95% CI from 1.21 to 1.86, log-rank *p* = 0.0002) (**Figure 3A**) and STAD (HR=0.77, 95% CI from 0.64 to 0.93, log-rank *p* = 0.0065) (**Figure 3B**). These results showed that the patients with low expression of CD68 were correlated with a better survival rate in COAD (**Figure 3A**). On the contrary, the patients with significant higher expression of CD68 have an improved survival rate in STAD (**Figure 3B**). There is also a significant difference in OS between patients with ESCA and LIHC (**Supplementary Figures S1A, B**), but this is contradictory to the expression level of CD68. There is no significant difference in OS between patients with PAAD (**Supplementary Figure S1C**). In brief, the expression level of CD68 impacted OS and could be served as prognostic marker in COAD and STAD.

### 3.4 Expression of CD68 was strongly positive correlated with six TIICs (B cells, CD4+ T cells, CD8+ T cells, macrophages, NK cells and cancer associated fibroblast) in DSC

**Figure 4:**
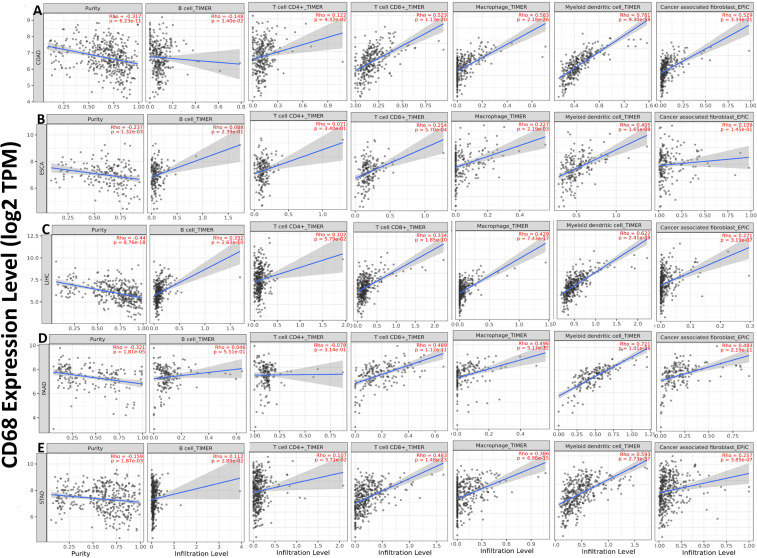
Correlation of CD68 expression with six TIICs in DSC. **(A)** CD68 expression has significant negative relation with tumor purity and B cell, significant positive correlation with tumor-infiltrating levels of CD4+ T cell, CD8+ T cell, macrophage, myeloid dendritic cell and cancer associated fibroblast in COAD. **(B)** CD68 expression has significant negative relation with tumor purity and significant positive correlation with tumor-infiltrating levels of B cell, CD4+ T cell, CD8+ T cell, macrophage, myeloid dendritic cell and has no relation with cancer associated fibroblast in ESCA. **(C)** CD68 expression has significant negative relation with tumor purity and significant positive correlation with tumor-infiltrating levels of B cell, CD4+ T cell, CD8+ T cell, macrophage, myeloid dendritic cell and cancer associated fibroblast in LIHC. **(D)** CD68 expression has significant negative relation with tumor purity and significant positive correlation with tumor-infiltrating levels of CD8+ T cell, macrophage, myeloid dendritic cell and cancer associated fibroblast, has no relation with B cell, CD4+ T cell in PAAD. **(E)** CD68 expression has significant negative relation with tumor purity, significant positive correlation with tumor-infiltrating levels of B cell, CD4+ T cell, CD8+ T cell, macrophage, myeloid dendritic cell and cancer associated fibroblast in STAD. Each dot represents a single tumor sample. P < 0.05 is considered as significant.

We explored the relationships between CD68 expression and six TIICs (B cells, CD4+ T cells, CD8+ T cells, macrophages, NK cells and Cancer associated fibroblast) focusing on COAD, ESCA, LIHC, PAAD and STAD. The results demonstrated that CD68 expression has significant negative relation with tumor purity and B cell, significant positive correlation with tumor-infiltrating levels of CD4+ T cell, CD8+ T cell, macrophage, myeloid dendritic cell and cancer associated fibroblast in COAD (**Figure 4A**). CD68 expression has significant negative relation with tumor purity and significant positive correlation with tumor-infiltrating levels of B cell, CD4+ T cell, CD8+ T cell, macrophage, myeloid dendritic cell and has no relation with cancer associated fibroblast in ESCA (**Figure 4B**). CD68 expression has significant negative relation with tumor purity and significant positive correlation with tumor-infiltrating levels of B cell, CD4+ T cell, CD8+ T cell, macrophage, myeloid dendritic cell and cancer associated fibroblast in LIHC (**Figure 4C**). CD68 expression has significant negative relation with tumor purity and significant positive correlation with tumor-infiltrating levels of CD8+ T cell, macrophage, myeloid dendritic cell and cancer associated fibroblast, has no relation with B cell, CD4+ T cell in PAAD (**Figure 4D**). CD68 expression has significant negative relation with tumor purity, significant positive correlation with tumor-infiltrating levels of B cell, CD4+ T cell, CD8+ T cell, macrophage, myeloid dendritic cell and cancer associated fibroblast in STAD (**Figure 4E**).

### 3.5 *CD68* expression has significant positive correlation with four common TICs (PDCD1, CTLA4, IDO1 and CD40) in DSC

**Figure 5:**
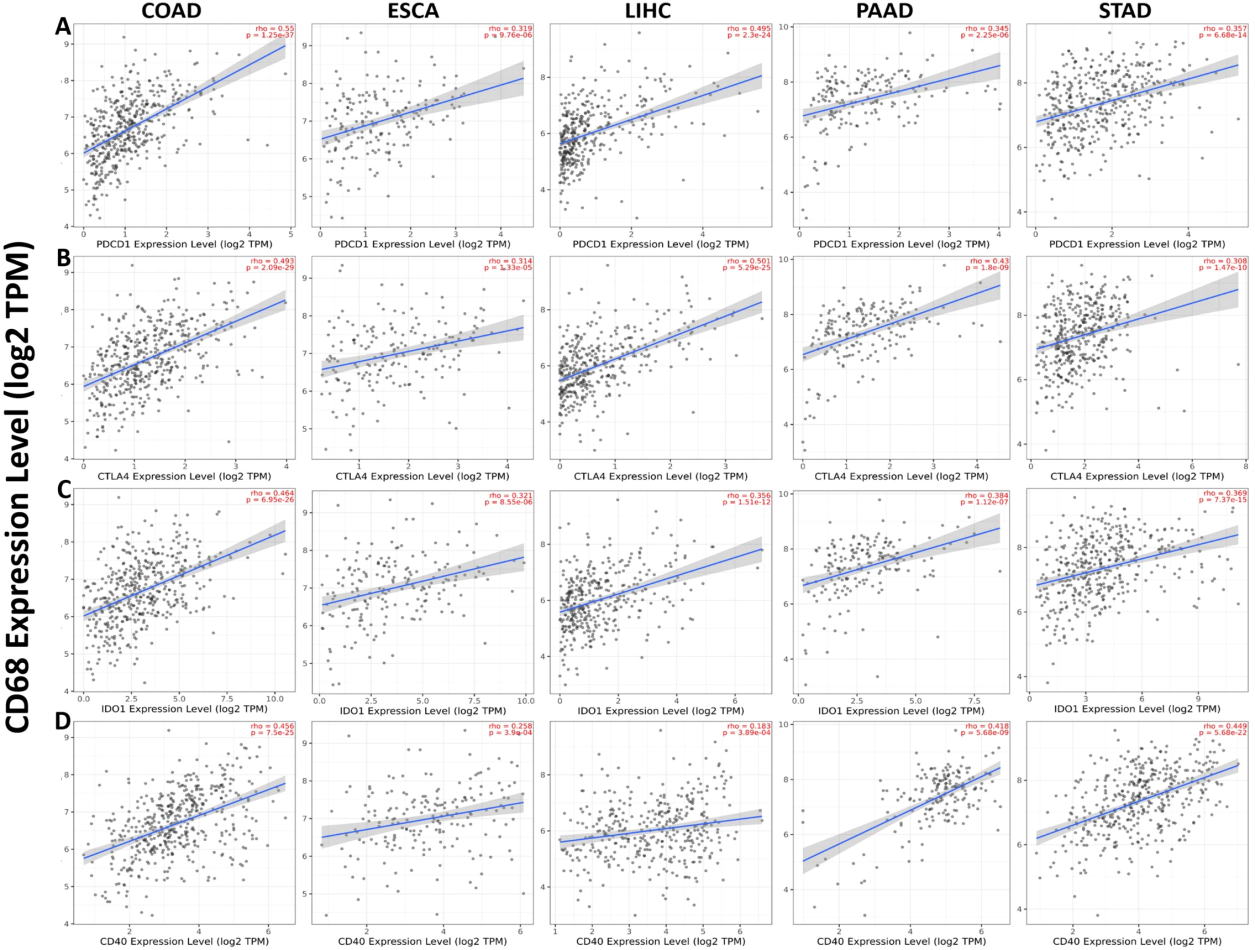
Correlation of CD68 expression with four common TICs (PDCD1, CTLA4, IDO1 and CD40) in DSC. **(A)** CD68 expression has significant positive correlation with PDCD1 in COAD (n=458), ESCA (n=185), LIHC (n=371), PAAD (n=179) and STAD (n=415). **(B)** CD68 expression has significant positive correlation with CTLA4 in COAD (n=458), ESCA (n=185), LIHC (n=371), PAAD (n=179) and STAD (n=415). **(C)** CD68 expression has significant positive correlation with IDO1 in COAD (n=458), ESCA (n=185), LIHC (n=371), PAAD (n=179) and STAD (n=415). **(D)** CD68 expression has significant positive correlation with CD40 in COAD (n=458), ESCA (n=185), LIHC (n=371), PAAD (n=179) and STAD (n=415). P < 0.05 is considered as significant.

We analyzed the correlation between CD68 expression with four common TICs (PDCD1, CTLA4, IDO1 and CD40) in COAD, ESCA, LIHC, PAAD and STAD. The results showed that *CD68* expression has significant positive correlation with PDCD1 in COAD, ESCA, LIHC, PAAD and STAD (**Figure 5A**). CD68 expression has significant positive correlation with CTLA4 in COAD, ESCA, LIHC, PAAD and STAD (**Figure 5B**). CD68 expression has significant positive correlation with IDO1 in COAD, ESCA, LIHC, PAAD and STAD (**Figure 5C**). *CD68* expression has significant positive correlation with CD40 in COAD, ESCA, LIHC, PAAD and STAD (**Figure 5D**).

### 3.6 mIF reveals distinct macrophage polarization and PD-L1 co-localization in COAD and STAD

**Figure 6:**
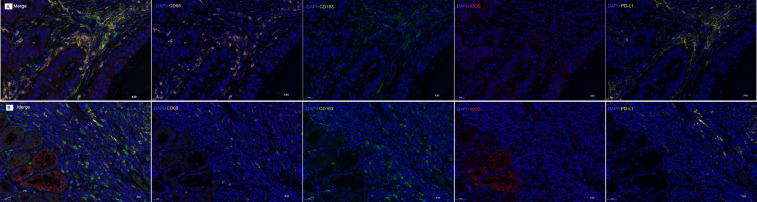
mIF Analysis of CD68+ Macrophage Polarization and PD-L1 Co-localization in COAD and STAD. **(A)** mIF images of COAD samples: Composite image (Merge) of all markers; DAPI (blue) +CD68 (pink); DAPI (blue)+CD163 (green); DAPI (blue)+iNOS (red); DAPI (blue)+PD-L1 (yellow). **(B)** mIF images of STAD samples: Composite image (Merge) of all markers; DAPI (blue)+CD68 (pink); DAPI (blue)+CD163 (green); DAPI (blue)+iNOS (red); DAPI (blue)+PD-L1 (yellow). Original magnification: 40×. Scale bars: 25μm.

Our mIF analysis revealed distinct patterns of macrophage polarization and PD-L1 co-localization between COAD and STAD specimens (**Figure 6**). In COAD tissues, CD68+ TAMs demonstrated significantly greater co-expression with the M2 marker CD163 compared to the M1 marker iNOS (*p*<0.001), with PD-L1 co-localization observed in 68.1%CD68+ cells, indicating a predominant M2-polarized macrophage infiltration (**Figure 6A**). In contrast, STAD specimens exhibited an inverse pattern, with CD68+ TAMs showing stronger association with iNOS than CD163 (*p*<0.001) and with PD-L1 co-expression in 65.2%CD68+cells, suggesting a dominant M1-like activation state in the STAD microenvironment (**Figure 6B**). These differential TAM polarization states - M2-dominant in COAD versus M1-dominant in STAD - provide a mechanistic basis for the observed tissue-specific prognostic significance of CD68 expression.

## 4 Discussion

The present study aimed to elucidate the expression patterns, prognostic significance, and clinical implications of CD68 in human DSC, as well as its correlations with TIICs and TICs. Our findings reveal that CD68 exhibits distinct expression profiles across different types of DSC, with significant implications for prognosis and immune modulation. These results suggest that CD68 may serve as a valuable prognostic biomarker and a potential therapeutic target in DSC.

Our analysis of CD68 mRNA and protein expression in DSC revealed tissue-specific patterns. CD68 mRNA expression was significantly elevated in ESCA and STAD compared to normal tissues, while it was reduced in COAD, LIHC, and PAAD. These findings align with previous studies suggesting that CD68 expression varies across cancer types and may reflect the heterogeneity of TAMs within the TME (5, 6). Interestingly, CD68 protein expression was significantly higher in COAD compared to adjacent normal tissues, but lower in ESCA, LIHC, PAAD, and STAD. This discrepancy between mRNA and protein levels may be attributed to post-transcriptional regulation or differences in macrophage activation states within the TME (9, 10).

The observed discrepancies between CD68 mRNA and protein expression levels across different DSC types may be explained by several biological mechanisms. First, post-transcriptional regulation, including mRNA stability, microRNA-mediated silencing, or alternative splicing, could account for the discordance between transcript abundance and protein levels. For instance, in LIHC and PAAD where CD68 mRNA was reduced but protein showed no significant change, there may be enhanced mRNA translation efficiency or reduced protein degradation compensating for lower transcript levels. Second, differences in cell type composition within tumor samples could contribute to these variations - while CD68 is primarily a macrophage marker, its expression may also occur in other myeloid cell populations that differ in proportion across cancer types.

The tumor microenvironment’s spatial and functional heterogeneity likely plays a crucial role in shaping these expression patterns. In COAD, where CD68 protein was elevated despite lower mRNA, this may reflect: (1) selective recruitment of CD68-high macrophage subsets to the tumor niche, (2) post-translational modifications stabilizing the protein, or (3) contributions from other CD68-expressing stromal cells. The nuclear localization of CD68 observed in STAD but no other cancers suggests tissue-specific processing or function of this protein, potentially through differential cleavage or trafficking mechanisms.

Microenvironmental factors such as hypoxia, extracellular matrix composition, or metabolic reprogramming could differentially regulate CD68 expression across tumor types. For example, the hypoxic cores of LIHC tumors might suppress CD68 translation while promoting protein stabilization in surviving macrophages. Additionally, the observed correlations with immune checkpoints may reflect functional crosstalk - CD68+ macrophages in different cancers may exhibit distinct capacities for PD-L1 induction or cytokine secretion that shape these relationships.

These findings underscore that CD68’s biological role depends on complex interactions between transcriptional regulation, protein handling, and tissue-specific microenvironmental factors.

CD68 protein expression demonstrated significant prognostic value in COAD and STAD. In COAD, low CD68 expression was associated with improved OS, suggesting that high levels of CD68 may promote tumor progression, possibly through increased macrophage infiltration and pro-tumorigenic signaling (16). Conversely, in STAD, high CD68 expression correlated with better survival outcomes, indicating a potential protective role of CD68 in this cancer type. This dual role of CD68 highlights its context-dependent functions in different DSC subtypes, which may be influenced by the specific TME and macrophage polarization states (6, 17). The polarization of macrophages into pro-inflammatory M1 or immunosuppressive M2 phenotypes (26) may explain the divergent prognostic roles of CD68 in COAD (poor prognosis) versus STAD (favorable prognosis). CD68+ macrophages in STAD may exhibit M1-dominant anti-tumor activity, while in COAD, they may adopt an M2-like, pro-tumorigenic state. The contradictory findings in ESCA and LIHC, where CD68 expression did not consistently correlate with survival outcomes, further underscore the complexity of CD68’s role in cancer progression.

Our study revealed strong positive correlations between CD68 expression and the infiltration levels of various TIICs, including B cells, CD4+ T cells, CD8+ T cells, macrophages, NK cells, and cancer-associated fibroblasts, across all DSC types. These findings suggest that CD68 may play a central role in modulating the immune landscape of DSC. The significant negative correlation between CD68 expression and tumor purity further supports the idea that CD68 is closely associated with immune cell infiltration rather than tumor cell density. This aligns with the established role of TAMs in shaping the TME and influencing tumor behavior through interactions with other immune cells (4, 5). The strong association between CD68 and macrophages is particularly noteworthy, as it reinforces the idea that CD68 serves as a reliable marker for TAMs in DSC (8, 9).

CD68 expression also showed significant positive correlations with four key immune checkpoints—PDCD1 (PD-1), CTLA4, IDO1, and CD40—in all DSC types. These immune checkpoints are critical regulators of immune responses and have emerged as promising targets for cancer immunotherapy (18). The strong association between CD68 and these checkpoints suggests that CD68 may influence immune evasion mechanisms in DSC, potentially through the modulation of TAM activity. For instance, the correlation between CD68 and PDCD1 (PD-1) implies that CD68-expressing macrophages may contribute to the immunosuppressive TME by upregulating PD-1/PD-L1 signaling, a well-known mechanism of immune evasion in cancer (18). Furthermore, CD40-CD154 interactions can modulate macrophage function and PD-1/PD-L1 signaling (27), suggesting that CD68+ macrophages may influence immune checkpoint regulation. Targeting these pathways could reprogram the TME and enhance immunotherapy efficacy in DSC. These findings highlight the potential of CD68 as a biomarker for identifying patients who may benefit from immune checkpoint blockade therapies.

The mIF data provide mechanistic insights into CD68’s context-dependent roles: In COAD, CD68+ macrophages predominantly exhibit an M2 phenotype (CD163+) with high PD-L1 co-expression, aligning with their pro-tumorigenic function and poor prognosis. In STAD, the M1-skewed (iNOS+) CD68+ population, despite PD-L1 co-localization, may retain anti-tumor activity through nitric oxide-mediated cytotoxicity, explaining the favorable prognosis. These findings highlight tumor-specific macrophage polarization as a key determinant of CD68’s prognostic value.

While this study provides valuable insights, several limitations should be acknowledged. First, the retrospective design relying on bioinformatics and immunohistochemistry analyses may introduce selection bias and preclude causal inference. Second, the single-institution origin of all clinical samples may limit generalizability due to potential regional variations in patient demographics, treatment protocols, and pathological evaluation standards. Third, while the TIMER database provides useful correlational data, its tumor purity adjustment cannot fully account for tumor microenvironment heterogeneity. Future studies incorporating single-cell RNA sequencing and functional assays are warranted to elucidate the precise mechanisms of CD68-mediated tumor immunity. Fourth, the prognostic significance of CD68 may vary across different digestive system cancer subtypes and stages, necessitating validation in larger, multi-center cohorts with standardized clinical annotations. Finally, our Spearman’s correlation analyses (significance threshold p < 0.05) did not include multiple testing correction due to the exploratory nature of this study, though we recommend false discovery rate (FDR) adjustment in future investigations.

## 5 Conclusion

In conclusion, our study establishes CD68 as both a prognostic biomarker and therapeutic target in DSC, with distinct clinical implications across cancer types. The M2-dominant/CD68+ phenotype in COAD associates with poor prognosis, while the M1-dominant/CD68+ profile in STAD correlates with better outcomes. These cancer-specific polarization patterns, along with CD68’s strong immune correlations, suggest tailored immunotherapy approaches - particularly combining polarization modulators (e.g., CSF-1R inhibitors) with PD-1 blockade - should be developed based on tumor-type macrophage profiles.

## Data Availability

The original contributions presented in the study are included in the article/**Supplementary Files**. Further inquiries can be directed to the corresponding authors.
