# Supplementary material for: CD68 as a multi-omic prognostic biomarker in digestive system cancers: correlations with tumor-infiltrating immune cells and immune checkpoints
Source: Front Immunol. 2025 Aug 21;16:1599677. doi: 10.3389/fimmu.2025.1599677 (PMC12408268; doi:10.3389/fimmu.2025.1599677)

Supplementary Materials

# Supplementary Table1

| **TCGA**  **Abbr.** | **Organ** | **Cancer type** | **All patients** | **Clinicopathological feature** | | | |
| --- | --- | --- | --- | --- | --- | --- | --- |
|  |  |  |  | Stage I | Stage II | Stage III | Stage IV |
| **COAD** | Colon | Adenocarcinoma | 24 | 6 | 5 | 7 | 6 |
| **ESCA** | Esophagus | Squamous Cell Carcinoma | 24 | 5 | 7 | 6 | 6 |
| **LIHC** | Liver | Hepatocellular carcinoma | 24 | 2 | 7 | 3 | 12 |
| **PAAD** | Pancreas | Invasive ductal carcinoma | 24 | 3 | 5 | 9 | 7 |
| **STAD** | Stomach | Tubular adenocarcinoma | 24 | 9 | 5 | 4 | 6 |

**Supplementary Figure 1.** **Kaplan-Meier survival curves comparing the high and low protein expression of CD68 in ESCA, LIHC and PAAD.** **A**, OS of ESCA (n=81), **B**, OS of LIHC (n=364). **C**, OS of PAAD (n=177). Red curve indicates patients with high expression of CD68, while black curve indicates patients with low expression of CD68. HRs with 95%CIs and log-rank P-values were calculated and shown in each curve. P < 0.05 was considered statistically significant.


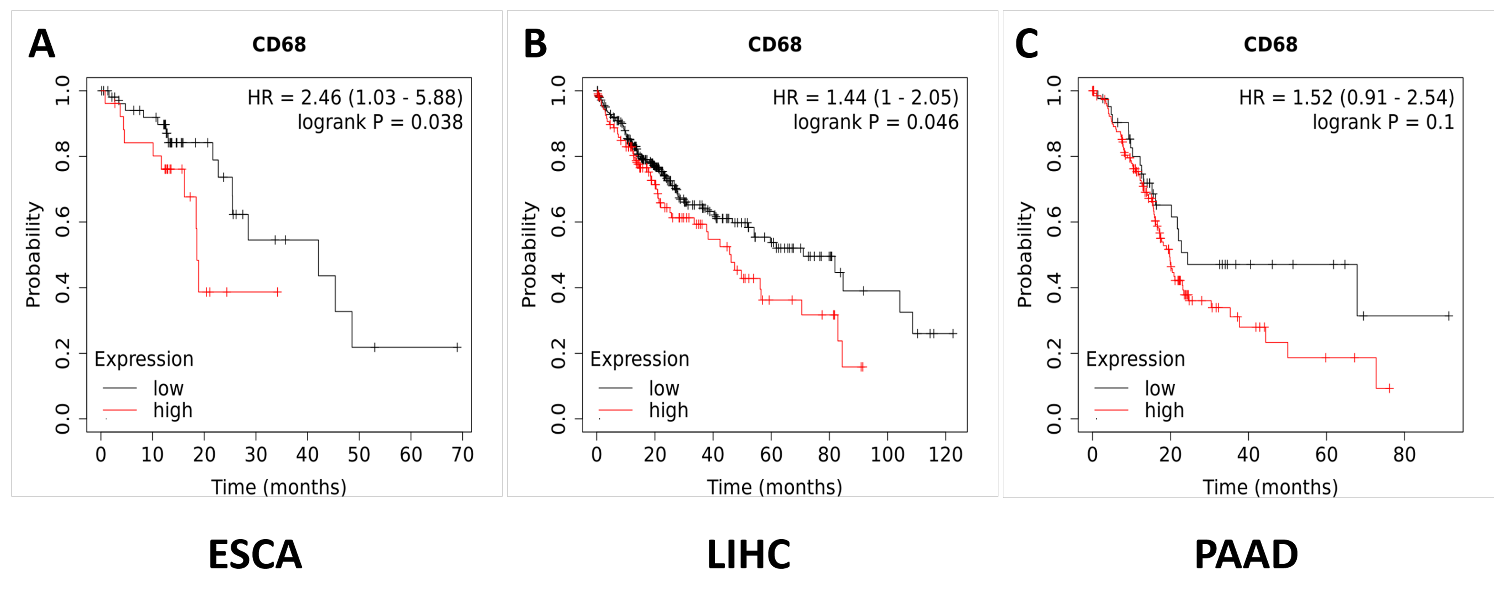

Supplement: Supplementary file 1 [file Table1.docx]
